# The Civic Hospital of Venice

**Published:** 1908-07-18

**Authors:** 


### EDITOR'S LETTER-BOX.

### THE CIVIC HOSPITAL OF VENICE.

Sir,—As I have some personal knowledge of the excellent Civic Hospital at Venice of which Mr. Conrad Thies gave a short sketch in your issue of July 4, you may, perhaps, allow me to add a note to that article. The hospital has done in the past, and, I believe, is still doing, a very good work, and is, like so many institutions dependent upon charity for a great part of their funds, not by any means a rich one. It takes in yearly several English patients—for the most part sailors. Thus on my several visits I found each time a patient who was a British subject. In every case these men spoke very highly of their treatment while in hospital, and in one case which I have in mind—that of a man with obscure liver trouble, probably of malignant nature—this was a particularly valuable testimony, as the man had been to two London and one Edinburgh hospital, and declared that if he had to choose between them all he would prefer the Civic Hospital. The hospital, as Mr. Thies has righly pointed out, is one of the sights of Venice. Its magnificent courtyard and fine library, and the Palazzo del Fratri portion are always open to the public, and are, I think, yearly visited by many score of English sight-seers. For examining other public structures in the city of the Doges a comparatively high charge is made; the Civic Hospital is free, and I would appeal to English visitors to support it by paying into the contribution box at least as much as they tip the porter who shows them round.

I may mention that the cases in the wards are very varied, and usually many interesting ones are to be found. The Italian colleagues are always truly helpful and courteous to the English graduate. and the vast amount of clinical materia[illegible] of great value to the professional visitor. Few, indeed [illegible]o have not spent a short time at the Ospedale Civico can have any idea of the opportunities for medical study Venice affords, especially for seeing varieties of that scourge of Italy—tuberculosis—and of some rare diseases, such as pellagra.

I am, etc.,
Graduate-Student.

Vienna, July 1908.

